# DO AGE-FRIENDLY COMMUNITIES PROMOTE OLDER ADULTS’ HEALTH?

**DOI:** 10.1093/geroni/igac059.2422

**Published:** 2022-12-20

**Authors:** Kyeongmo Kim, Denise Burnette, Thomas Buckley, Seon Kim

**Affiliations:** Virginia Commonwealth University, Richmond, Virginia, United States; Virginia Commonwealth University, Richmond, Virginia, United States; University of Pittsburgh, Pittsburgh, Pennsylvania, United States; Virginia commonwealth university, Richmond, Virginia, United States

## Abstract

The WHO developed the concept of age-friendly environments that contribute to older adults' better health and well-being. However, research on the effects of age-friendly communities (AFCs) on their health is limited. As AFC initiatives grow, it will be essential to examine whether older adults who live in an AFC have better health than those in other environments. This study uses data from the 2017 AARP AFC Surveys and the AARP Livability Index to assess whether AFCs promote the health of older adults. We analyze data for 3,027 adults aged 65 and older who reside in 262 zip code areas. Following AARP guidelines, we allocated the sample into two groups: the AFC group (livability score of 51+; n=2,364) and the non-AFC group (score≤50, n=663). The outcome variable was self-rated health (M=3.47; SD=1.09; range: 1-5). Findings employing an inverse probability weighting approach showed that older adults who lived in an AFC had better self-rated health than those in a non-AFC (b=0.08, p=.027). Compared to non-Hispanic whites, Black and Hispanic older adults reported worse self-rated health. Oldest old individuals had better health status than young-old adults. Consistent with previous literature, higher education, and frequent social interaction were associated with better self-rated health. The study adds to the growing literature on the role of age-friendly environments in older adults’ health and suggests directions for future research. Since living in an AFC can promote the well-being of older adults, policymakers and practitioners should continue to build high-quality, accessible built and social environments.

